# Social instability stress differentially affects amygdalar neuron adaptations and memory performance in adolescent and adult rats

**DOI:** 10.3389/fnbeh.2014.00027

**Published:** 2014-02-03

**Authors:** Sheng-Feng Tsai, Tung-Yi Huang, Chia-Yuan Chang, Yuan-Chang Hsu, Shean-Jen Chen, Lung Yu, Yu-Min Kuo, Chauying J. Jen

**Affiliations:** ^1^Department of Physiology, National Cheng Kung UniversityTainan, Taiwan; ^2^Institute of Basic Medical Sciences, National Cheng Kung UniversityTainan, Taiwan; ^3^Department of Engineering Science, National Cheng Kung UniversityTainan, Taiwan; ^4^Advanced Optoelectronic Technology Center, National Cheng Kung UniversityTainan, Taiwan; ^5^Institute of Behavioral Medicine, National Cheng Kung UniversityTainan, Taiwan; ^6^Department of Cell Biology and Anatomy, National Cheng Kung UniversityTainan, Taiwan

**Keywords:** adolescent, dendritic spine, memory, neuroplasticity, TrkB

## Abstract

Adolescence is a time of developmental changes and reorganization in the brain. It has been hypothesized that stress has a greater neurological impact on adolescents than on adults. However, scientific evidence in support of this hypothesis is still limited. We treated adolescent (4-week-old) and adult (8-week-old) rats with social instability stress for 5 weeks and compared the subsequent structural and functional changes to amygdala neurons. In the stress-free control condition, the adolescent group showed higher fear-potentiated startle responses, larger dendritic arborization, more proximal dendritic spine distribution and lower levels of truncated TrkB than the adult rats. Social instability stress exerted opposite effects on fear-potentiated startle responses in these two groups, i.e., the stress period appeared to hamper the performance in adolescents but improved it in adult rats. Furthermore, whilst the chronic social stress applied to adolescent rats reduced their dendritic field and spine density in basal and lateral amygdala neurons, the opposite stress effects on neuron morphology were observed in the adult rats. Moreover, stress in adolescence suppressed the amygdala expression of synaptic proteins, i.e., full-length TrkB and SNAP-25, whereas, in the adult rats, chronic stress enhanced full-length and truncated TrkB expressions in the amygdala. In summary, chronic social instability stress hinders amygdala neuron development in the adolescent brain, while mature neurons in the amygdala are capable of adapting to the stress. The stress induced age-dependent effects on the fear-potentiated memory may occur by altering the brain-derived neurotrophic factor (BDNF)-TrkB signaling and neuroplasticity in the amygdala.

## Introduction

Adolescence, a transitional stage from childhood to adulthood, in humans or animals is a particular period of life in which significant physiological and psychological changes occur. Intuitively such changes appear to arise from structural and functional alterations in the brain. In this phase of rapid growth, the brain reorganizes the synaptic connections and many neurotransmitter systems, and is more readily shaped by environmental factors, such as stress (Spear, 2000). Stressful experiences in adolescence modify behavioral responses to external stimuli in adulthood, e.g., the learning and memory of stressful events (Morrissey et al., 2011).

The amygdala is an essential component of the neural circuitry governing stress responses and fear memory (Roozendaal et al., 2009). It contains a heterogeneity of distinct nuclei, differing by cell type, density, neurochemical composition, and connectivity (Rodrigues et al., 2009). Both basal and lateral nuclei of amygdala (BLA) are generally considered as the sensory gateways of the amygdala, while the central nucleus serves as an important output region that controls behavioral, autonomic, and endocrine responses via projections to downstream areas, such as the hypothalamus (Swanson, 2003; LeDoux, 2007). Notably, external aversive stimuli often lead to the activation of the hypothalamic-pituitary-adrenal (HPA) axis and the release of stress hormones. Lastly, stress hormones in the amygdala regulate the formation of fear memory (Roozendaal et al., 2009).

A wide body of evidence has shown that stressful experiences positively alter the amygdalar structure and function in adult animals. For examples, both acute and chronic stressors improve the performances in amygdala-related cued-fear conditioning (Conrad et al., 1999; Hui et al., 2006) and increased the dendritic arborization and spine density of neurons in the BLA (Vyas et al., 2002; Mitra et al., 2005). Amygdalar neuroplasticity-related markers, the brain-derived neurotrophic factor (BDNF) in particular, are upregulated by chronic stress and thus contribute to the facilitation of aversive learning and memory (Morrissey et al., 2011; Lakshminarasimhan and Chattarji, 2012). However, relatively little is known about the brain structural and functional changes that may underlie the stress effects in adolescent animals. A pioneering study shows that stress hampers contextual and auditory fear conditioning in adolescent, but not adult rats (Morrissey et al., 2011). It is plausible to hypothesize that stress in adolescence may negatively affect the amygdalar structure and function. However, scientific evidence in support of this hypothesis is still limited. This study is designed to compare the effects of chronic social instability stress on the amygdalar functions and structure in adolescent and adult rats.

Male rats at 4 (adolescent) and 8 (adult) weeks of age were subjected to 5 weeks of social instability stress. A fear-potentiated startle (FPS) paradigm was used to evaluate the functional performance of amygdala-related learning and memory (Campeau and Davis, 1995; Davis, 2006). Stress-induced changes in neuroplasticity were determined by neuronal morphology in the BLA and the expression levels of BDNF, its receptor, TrkB, and two synaptic fusion proteins, synaptotagmin I (Syt I) and SNAP-25, in the amygdala of both age groups.

## Materials and methods

### Animals

Male Sprague Dawley rats were obtained from the Laboratory Animal Center of National Cheng Kung University at 3 (adolescent) or 7 (adult) weeks of age. Rats of the same age were housed five per cage and kept on a 12–12 h light-dark cycle with free access to rat chow and water. Sixteen adolescent rats (7 control, 9 stress) and 20 adult rats (10 control, 10 stress) were used for the FPS study. Five rats per group were used for dendritic arbor and spine analyses. Twenty adolescent rats (10 control, 10 stress) and 14 adult rats (7 control, 7 stress) were used for protein quantifications. The body weight (*n* = 10 rats in each group) was measured once per week and the amounts of food consumption (*n* = 4 cages of rats in each group) were recorded every day. The measurements of body weight and food intake were performed before the daily stress or handling. All experimental protocols were performed according to National Institutes of Health Guideline for animal research (Guide for the Care and Use of Laboratory Animals) and approved by the National Cheng Kung University Institutional Animal Care and Use Committee (IACUC number 98162). All efforts were made to minimize the number of animals used and any suffering.

### Chronic social instability stress

Adolescent and adult rats were randomly divided into stress and control groups. The stress protocol was modified from a previous report (Morrissey et al., 2011). After 1 week acclimation in the laboratory, the stress group received 5 week daily social instability stress, i.e., rats were individually immobilized in cone-shaped wire mesh for 1 h and returned to a new cage with different roommates by switching two out of five rats. Control rats received normal handling for 2 min/day. Although, by the end of the stress treatment the adolescent rats turned into adulthood (9-week-old), they were still referred to as the “adolescent” group to avoid confusion.

### Fear-potentiated startle (FPS)

The behavioral apparatus and procedures of FPS were the same as described previously (Hsu et al., 2012). Briefly, 4 days before the completion of 5-week stress treatments, rats were prepared for the FPS task. The procedures of FPS were carried out in two separate but identical startle reflex systems (SR-Lab, San Diego Instruments, San Diego, CA, USA). The acoustic startle stimulus was a 50 ms white noise at an intensity of 95 dB. The visual conditioned stimulus (CS) was a 3.7 s light and the unconditioned stimulus (US) was a 0.4 mA footshock with duration of 0.5 s.

In the beginning, all rats were placed in a training cabinet for 10 min and returned to their home cages for three consecutive days. On the following two days, they were placed in the same chamber to obtain baseline startle values via pre-exposure to 30 acoustic stimuli (noise bursts, 95 dB, 50 ms duration, with a 30 s interstimulus interval). On the day of fear conditioning (24 h after final stress or normal handling), each animal was brought to the room, allowed to habituate, and placed in the previously exposed cabinet. The CS-US pairing began after a 5 min acclimation period in this training cabinet. In the training session, rats in the cabinet received eight co-terminated light-footshock (CS-US) pairings with an inter-trial interval of 3 ~ 5 min.

In the testing session, rats were placed in a different cabinet 24 h later, and were pre-exposed to 30 noise bursts (95 dB, 50 ms, 30 s inter-stimulus interval) first. Then they were tested for the fear-potentiated startle, a process which involved 10 noise bursts alone (dark-noise trials) and 10 noise bursts presented 3.2 s after the onset of 3.7 s light (light-noise trials). The 20 trials were given randomly. FPS (%) was defined as (Startle^light^ − Startle^dark^) / Startle^dark^ × 100.

### Single-neuron labeling

One day after the completion of the 5 week stress treatments, rats were anesthetized with urethane (1.5 g/kg, i.p.). Anesthetized animals were perfused from the left ventricle with 300 ml artificial cerebrospinal fluid (117 mM NaCl, 4.7 mM KCl, 2.5 mM CaCl_2_, 11 mM glucose, 1.2 mM MgCl_2_, 25 mM NaHCO_3_, 1.2 mM NaH_2_PO_4_, pH 7.4) and followed by 4% paraformaldehyde (in 0.1 M phosphate buffer, pH 7.4; 300 ml/400 g animal weight). The brain was dissected and post-fixed with 4% paraformaldehyde at 4°C for 3 h.

Single-neuron labeling with the fluorescent dye lucifer yellow was performed according to methods described previously (Huang et al., 2012). Briefly, coronal sections (200 µm thickness) were viewed under differential interference contrast optics to identify neuron soma in the BLA. The neuron soma, located in the middle layer of the brain slice, was impaled with an intracellular electrode filled with saturated lucifer yellow solution. The fluorescent dye was delivered iontophoretically to fill the entire neuron. After labeling, the slice was dehydrated in saturated sucrose solution and then mounted with the medium consisting of glycerol (80%) and 20 mM sodium carbonate (20%).

### Morphological analysis of basal and lateral nuclei of amygdala (BLA) neurons

Morphological analysis of BLA neurons were performed as described previously (Lin et al., 2012). Briefly, a conventional fluorescence microscope was used to trace the labeled dendrites. The dendritic field of the fluorescent labeled basal and lateral amygdala neuron was measured by Sholl analysis, which determined the branch and length distribution of dendrites using 10 µm increment concentric rings. The dendrites were traced under a 40x objective lens and then analyzed up to 30 concentric rings. The dendritic spines were examined under a custom-made two-photon laser scanning microscope; this image system consisted of a femtosecond laser (Tsunami, Spectra-Physics, Irvine, CA, USA), an acousto-optic modulator (23080-x-1.06-LTD, NEOS Technologies, Melbourne, FL, USA), a galvanometer scanner (6215H, Cambridge, MA, USA), an inverted optical microscope (Axiovert 200, ZEISS, Oberkochen, Germany), a three-axis motorized stage (ProScanTMII, Prior Scientific Inc., Rockland, MA, USA), a *z*-axis piezoelectric nano-positioning stage (Nano-F100, Mad City Laboratories, Madison, WI, USA), and a photomultiplier tube (H5783P, Hamamatsu, Shizuoka, Japan). The dendritic spine of BLA neuron was observed using a 63x objective lens (*NA* = 1.4, Oil DIC, Plan-APOCHROMAT, ZEISS, Oberkochen, Germany). A projected image (50 µm × 50 µm) was reconstructed from consecutive optic sections (*z*-axis depth 20 µm, 0.5 µm each section). We used the cell counter plugin of ImageJ software to manually count the spine number.

The spine densities, expressed as spines/µm, were quantified along the primary dendrite of BLA neurons. After *z*-axis projection (3-dimension converted to 2-dimension), an average length of 10 µm in the 1st dendrite and 25 µm in the 2nd and 3rd dendrites were used for spine number quantification. Only those dendrites with distances of *z*-axis (depth) less than 10 µm were used in this study. Five neurons in five brain slices obtained from each animal were labeled and measured in regions of interest. Five animals in each group were analyzed.

### Immunoblotting

Immunoblotting of amygdalar TrkB, Syt I, and SNAP 25 was performed as described previously (Lin et al., 2012). Ten rats in each group were used. Relative protein levels against β-actin were estimated by immunoblotting using the following primary antibodies: mouse monoclonal antibody against TrkB (1:5000 dilution; 610102, Becton Dickinson, Franklin Lakes, NJ, USA), mouse monoclonal antibody against Syt I (1:10,000 dilution; SYA-130, Stressgen, Victoria, BC, Canada), and mouse monoclonal antibody against SNAP-25 (1:10,000 dilution; VAM-SV012, Stressgen, Victoria, BC, Canada).

### Statistical analysis

Data were expressed as mean ± SEM. The body weight, food intake, and dendritic field of individual neurons (Sholl analysis) was analyzed by repeated measured (mixed-model) ANOVAs with age and concentric rings (increasing diameters of 10 µm) as within-subject factor and stress as between subject factor. Results of fear-potentiated startle, dendritic spine density, and amygdala protein levels were analyzed by two-way ANOVA followed by Bonferroni’s *post hoc* test.

## Results

### Effects of stress on body weight and food intake in adolescent and adult rats

**Figure 1:**
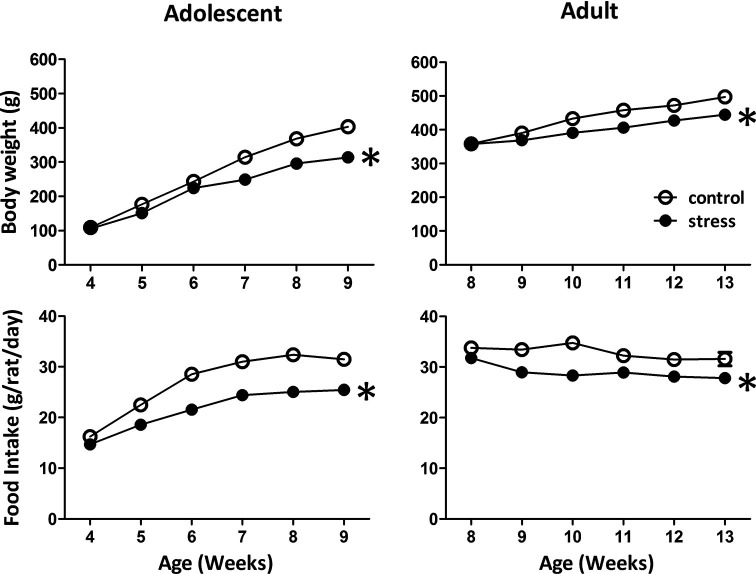
**Effects of stress on body weight and food intake in adolescent and adult rats.** The body weight and food intake were monitored to evaluate the stress responses in adolescent and adult rats. * significantly different between control and stress groups, repeated measured ANOVAs.

Reduced body weight gain and food consumption are typical stress responses in rodents (Harris et al., 1998). Exposing adolescent rats to chronic social instability stress caused significant reductions in body weight gain (*F* = 168.9, *d.f.* 1/90, *p* < 0.001; Figure 1) and food consumption (*F* = 119.7, *d.f.* 1/90, *p* < 0.001; Figure 1). Similar effects were observed in adult rats exposed to stress (body weight gain: *F* = 5.4, *d.f*. 1/30, *p* < 0.05; food intake: *F* = 48.7, *d.f*. 1/30, *p* < 0.001; Figure 1). These data suggested that the 5-week social instability was an effective stress paradigm for both adolescent and adult rats.

### Effects of stress on the performances of fear-potentiated startle (FPS) task in adolescent and adult rats

**Figure 2:**
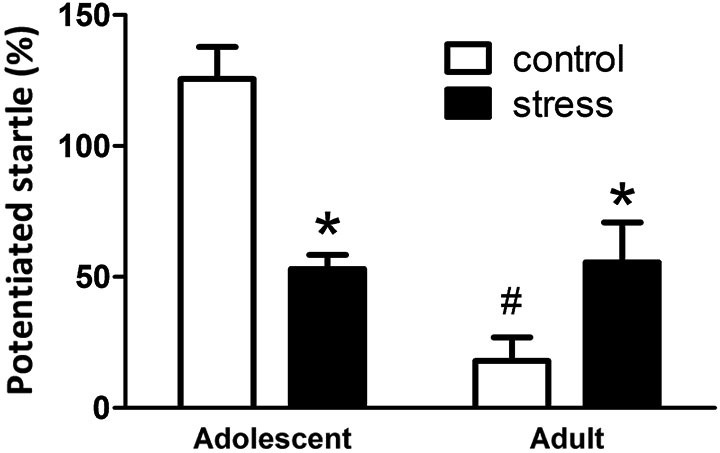
**Effects of stress on the performance of fear-potentiated startle task in adolescent and adult rats.** The same fear-potentiated startle task was used to test the amygdala-related learning and memory in adolescent and adult rats. * Significantly different between control and stress groups; ^#^** significantly different between adolescent control and adult control groups, two-way ANOVA followed by Bonferroni’s *post hoc* test.

At the end of the 5 weeks of stress treatment, the adolescent rats turned into 9-week-old (still named adolescent group) and the adult rats turned into 13-week-old. The 9-week-old adolescent control rats showed higher light-induced FPS responses than the 13-week-old adult controls (Figure 2, Adolescent vs. Adult control, *t* = 7.2, *d.f*. = 15, *p* < 0.001). Five weeks of social instability stress reduced the FPS scores in adolescent rats, whereas, the same stress paradigm increased the FPS in adult rats (*F* = 21.4, *d.f*. 1/32, *p* < 0.001; Figure 2). Thus, chronic social instability stress appeared to induce opposite effects on the amygdala-related learning and memory between adolescent and adult rats.

### Effects of stress on basal and lateral nuclei of amygdala (BLA) neuron morphology in adolescent and adult rats

**Figure 3:**
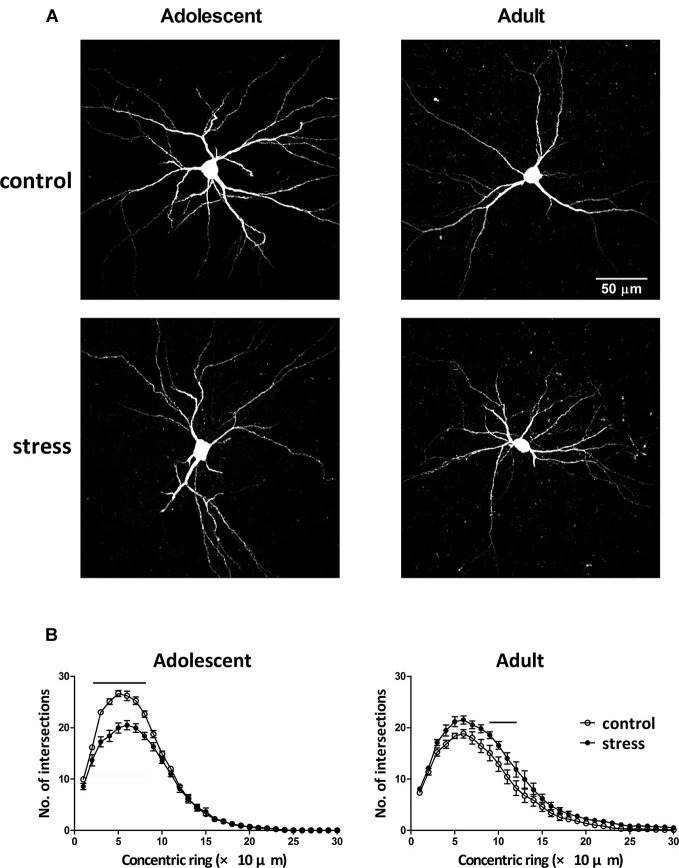
**Effects of stress on the dendritic field of BLA neurons in adolescent and adult rats. (A)** Representative two-photon laser scanning micrographs revealed the dendritic arbor of BLA neurons in adolescent and adult rats with (stress) or without (control) chronic social instability stress treatment. **(B)** Morphometric results were presented by the Sholl diagram. Bars indicate significant differences between stress and control groups in number of intersections with the concentric rings, repeated measured ANOVAs followed by Bonferroni’s *post hoc* test.

Neuronal morphology was revealed by injecting a fluorescent dye lucifer yellow into the soma of individual BLA neurons (Figure 3A). By comparing the results of Sholl analysis in two different ages (i.e., 9- vs. 13-week-old) of stress-free rats, the BLA neurons of 13-week-old controls had less dendritic intersections with concentric rings, especially in the range of 20–90 µm, than that of 9-week-old control rats (Figure 3B, adolescent control vs. adult control; *F* = 99.5, *d.f*. 1/240, *p* < 0.001). Besides, the length of the longest dendrite increased considerably during this developmental period (184.8 ± 10.7 µm vs. 236.4 ± 15.3 µm, 9-week-old controls vs. 13-week-old controls, *p* < 0.05). Five weeks of chronic social instability stress in adolescent rats reduced the dendritic field of BLA neurons. Mixed model ANOVA showed significant effects of stress on the number of dendritic intersections with concentric rings (Figure 3B, left panel; *F* = 119.3, *d.f*. 1/240, *p* < 0.001), especially in the range of 20 µm to 80 µm. In contrast, the dendritic field of BLA neurons in adult rats was augmented by stress (Figure 3B, right panel; *F* = 72.8, *d.f*. 1/240, *p* < 0.001), with more dendritic intersections with concentric rings between 90 µm and 120 µm than the control group.

**Figure 4:**
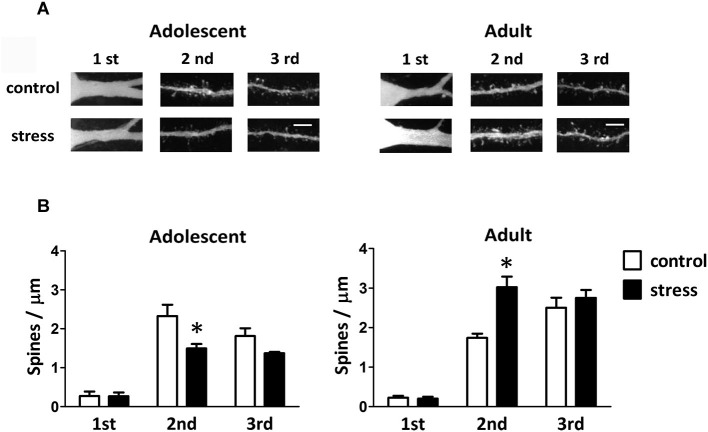
**Effects of stress on the spine density of BLA neurons in adolescent and adult rats. (A)** Representative two-photon laser scanning micrographs revealed spines located at different orders of dendrites of the BLA neurons in adolescent and adult rats with (stress) or without (control) chronic social instability stress treatment. Scale bar = 5 µm. **(B)** Quantitative results of the spine density at different order dendrites. * Significantly different between respective control and stress groups, two-way ANOVA followed by Bonferroni’s *post hoc* test.

Single-neuron labeled also allowed us to monitor the dendritic spines using two-photon laser scanning microscopy (Figure 4A). When compared to the 9-week-old control rats, the spine distributions of the 13-week-old control rats were more concentrated in the distal dendrites (Figure 4B; adolescent control: 2nd vs. 3rd dendrite, *p* = 0.197; adult control: 2nd vs. 3rd dendrite, *p* < 0.05). The social instability stress reduced the spine density in 2nd dendrites of the BLA neurons in adolescent rats, whereas it augmented the spine density in secondary dendrites in adult rats (Figure 4B). The proportions of different types of spines (i.e., thin, stubby and mushroom) in either adolescent or adult group were not altered by the social instability stress (data not shown).

### Effects of stress on the amygdalar expression of neuroplasticity-related proteins in adolescent and adult rats

**Figure 5:**
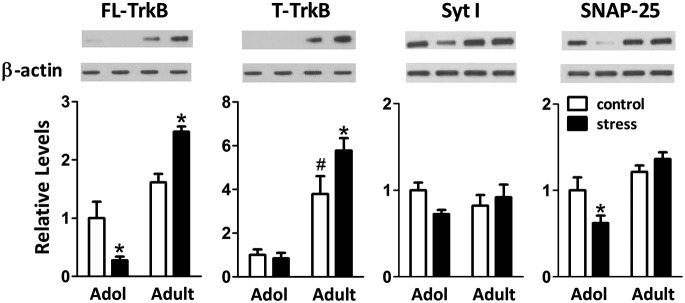
**Effects of stress on the levels of neuroplasticity-related proteins in the amygdala of adolescent and adult rats.** Representative immunoblotting micrographs are shown on the upper panels, whereas quantitative results are shown in the lower panels. β-actin was used as the internal control. * Significantly different between respective control and stress groups, unpaired *t*-test; ^#^** significantly different between adolescent control and adult control groups, two-way ANOVA followed by Bonferroni’s *post hoc* test.

When 9-week-old rats turned into 13-week-old under unstressed conditions, we noticed an elevation of the levels of truncated TrkB (T-TrkB)(*t* = 3.7, *d.f*. = 15, *p* = 0.002), but not full-length TrkB (FL-TrkB)(*t* = 1.7, *d.f*. = 15, *p* = 0.110), Syt I (*t* = 1.2, *d.f*. = 15, *p* = 0.243) or SNAP-25 (*t* = 1.1, *d.f*. = 15, *p* = 0.288) in the amygdala (Figure 5). Five weeks of chronic social instability stress induced different effects on the amygdalar levels of FL-TrkB (Interaction: *F* = 17.8, *d.f*. 1/30, *p* < 0.001), T-TrkB (Interaction: *F* = 5.3, *d.f*. 1/30, *p* = 0.029) and SNAP-25 (Interaction: *F* = 5.2, *d.f*. 1/30, *p* = 0.030), but not Syt I (Interaction: *F* = 3.6, *d.f*. 1/30, *p* = 0.069), between adolescent and adult rats. The stress down-regulated the amygdalar levels of FL-TrkB and SNAP-25, but not T-TrkB or Sty I, in the adolescent rats; whereas, the same stress up-regulated the expressions of amygdalar FL-TrkB and T-TrkB without changing the expression of Syt I and SNAP-25 in the adult rats (Figure 5). These results indicated that chronic social instability stress exerted differential effects on the expression of amygdalar neuroplasticity-related proteins in two age groups.

## Discussion

This study is designed to compare the functional and structural changes in amygdala between adolescent and adult experience of chronic social instability stress. Our results showed that the same stress paradigm applied to rats of different ages differentially affected their amygdala-related parameters, i.e., learning and memory performance, local neuron morphology and protein expression. In adolescent rats, chronic social instability suppressed the performance of fear-potentiated startle, reduced the dendritic arborization and spine density, and down-regulated the expressions of neuroplasticity-related proteins in the amygdala. This stress led to almost completely opposite consequences when applied to adult rats. Our results, at molecular and cellular levels, demonstrate that adolescents are more vulnerable to chronic stress than adults. These results also confirm previous findings that, in adult rats, chronic stress enhances the cued fear conditioning, increases the morphological complexity of amygdalar neurons, and up-regulates the BDNF-TrkB signal pathway (Conrad et al., 1999; Vyas et al., 2002; Mitra et al., 2005; Lakshminarasimhan and Chattarji, 2012).

Long term encoding of experience-related neural activity is capable of changing the neuronal structure and number of spine synapses, the so-called neuronal adaptation. During development and in adulthood, the size and number of dendrites and spines can be modified by many stimuli, such as learning, sensory experience and hormones, etc. Changes in the neuronal structures usually couple with functional changes of the connected neural networks. Our findings that the chronic stress paradigm applied to the adolescent rats reduced both dendritic field and spine density in the BLA neurons explains why stressed adolescent animals showed relatively poor FPS performance. Along the same line, the same stress-induced increases on the dendritic arbor and spine density of the BLA neurons in the adult rats also paralleled with the enhancement of FPS performance.

The opposite effects on the dendritic arbor and spine density of BLA neurons in the adolescent and adult rats induced by chronic social instability stress may be explained by the stress-induced differential expressions of FL-TrkB. BDNF-TrkB signaling is known to regulate dendritic growth in developing stage and neuroplasticity in adulthood (McAllister et al., 1995; Minichiello, 2009). A higher expression level of FL-TrkB has been linked to the addition of short dendritic branches in the regions proximal to the cell body of the layer VI cortical pyramidal neuron (Yacoubian and Lo, 2000). In agreement with these observations, we also found a paralleling relationship between the levels of FL-TrkB and the numbers of short dendrite and dendritic spines. Notably, our measured spine density of secondary dendrites of the BLA neurons in adult unstressed rats (1.7 ± 0.1 spines/µm) was considerably higher than that reported in a previous study (about 0.5 spines/µm) (Mitra et al., 2005). This discrepancy may be attributed to the technical advancements in staining and imaging. In that prior study, Golgi staining was used to visualize the density and morphology of dendritic spine under optical microscope, which is a method unable to capture the spine images located just above and below the dendritic trunk. In this study however, two-photon laser scanning system was employed which generated images from a stack of high-resolution optical sections (usually 0.5 µm apart), thus allowing identification of minute spines located just above and below the dendrite trunk.

Our experimental design also allowed us to determine the age-dependent changes in amygdala of stress-free rats. We found that adolescent rats exhibited much more pronounced FPS responses than the adult rats. As many age-associated factors (e.g., sensory and motor sensitivities and stress hormone responses) could potentially influence the FPS outcomes, direct comparisons of the FPS performances between young and mature animals may be misleading. However, we did observe an age-dependent change in the dendritic arbor of BLA neuron by comparing the results obtained from 9-week-old to 13-week-old control rats. We found that, as rats matured from 9 to 13 weeks of age, the dendritic arborization retracted accompanied by dendrite elongation and increase of spine density in the distal dendrite (i.e., 3rd order branch) of the BLA neurons. These results indicate that the BLA neurons are still in a pruning stage (a process containing reduction and reorganization of neural connections) during the early adulthood of rats. Furthermore, these findings also suggest that chronic stress might affect the pruning process during the BLA neuron maturation. Although at first glance chronic social instability stress-induced reduction of dendrite branch may seem to enhance the pruning process in the BLA neuron of the adolescent rat, results of lack of dendrite elongation, decrease of spine density in the distal dendrite and reduction of FL-TrkB all suggest that chronic social instability stress disturbs the normal growth of BLA neurons in the adolescent rats.

While an age-dependent increase in the levels of FL-TrkB was evident in the amygdala, we also noticed an age-dependent increase in the levels of T-TrkB in this region. The latter results are consistent with the findings that T-TrkB expression levels are the lowest in prenatal stage and dramatically increase as animals reach adulthood (Silhol et al., 2005). T-TrkB is translated from the same gene as FL-TrkB with alternative splicing that removes the exons encoding for the intracellular tyrosine kinase domain. Hence, T-TrkB can bind to and internalize BDNF, but it does not undergo autophosphorylation to function as a true tyrosine kinase receptor (Fenner, 2012). Over-expression of T-TrkB in the rat amygdala has been shown to, via dominant-negative manipulation of the BDNF-TrkB signaling, impair fear conditioning as assessed by the FPS task (Rattiner et al., 2004). However, by working together with FL-TrkB, T-TrkB is also known to modulate the activity of BDNF. Unlike FL-TrkB that increases the numbers of short dendrites in the regions proximal to the cell body, T-TrkB has been demonstrated to induce the net extension of dendrites in regions more distal to the soma (Yacoubian and Lo, 2000). Although the detailed mechanism for the FL-TrkB/T-TrkB-associated rearrangement of cytoskeleton and dendritic arbor remains unclear, it is reasonable to hypothesize that, in addition to their expression levels, the ratio of FL-TrkB:T-TrkB may also play a role in fine tuning the dendritic arbor of the BLA neurons during maturation.

In summary, this study analyzed the effects of chronic social instability stress on amygdala of adolescence and adulthood. Our results, at behavioral, morphological and molecular levels, demonstrated that the same stress paradigm has differential (opposite) effects on the function of amygdala at these two ages. We also found that the stress-induced age-dependent effects on the fear-potentiated memory possibly occur via the BDNF-TrkB signaling and neuroplasticity in the amygdala. This study also calls attention to the differential age effect, especially during the critical developmental stage, on the neuronal response to certain stimuli.

## Conflict of interest statement

The authors declare that the research was conducted in the absence of any commercial or financial relationships that could be construed as a potential conflict of interest.
